# Riociguat versus sildenafil on hypoxic pulmonary vasoconstriction and ventilation/perfusion matching

**DOI:** 10.1371/journal.pone.0191239

**Published:** 2018-01-24

**Authors:** Virginia Chamorro, Daniel Morales-Cano, Javier Milara, Bianca Barreira, Laura Moreno, María Callejo, Gema Mondejar-Parreño, Sergio Esquivel-Ruiz, Julio Cortijo, Ángel Cogolludo, Joan A. Barberá, Francisco Perez-Vizcaino

**Affiliations:** 1 Departamento de Farmacología. Facultad de Medicina, Universidad Complutense de Madrid, Madrid, Spain; 2 Ciber Enfermedades Respiratorias (Ciberes), Madrid, Spain; 3 Instituto de Investigación Sanitaria Gregorio Marañón (IISGM), Madrid, Spain; 4 Dept of Pharmacology, Faculty of Medicine, University of Valencia, Valencia, Spain; 5 Clinical Research Unit (UIC), University General Hospital Consortium, Valencia, Spain; 6 Department of Pulmonary Medicine, Hospital Clínic-Institut d'Investigacions Biomèdiques August Pi i Sunyer (IDIBAPS), Universitat de Barcelona, Barcelona, Spain; Stanford University, UNITED STATES

## Abstract

**Introduction:**

Current treatment with vasodilators for pulmonary hypertension associated with respiratory diseases is limited by their inhibitory effect on hypoxic pulmonary vasoconstriction (HPV) and uncoupling effects on ventilation-perfusion (V’/Q’). Hypoxia is also a well-known modulator of the nitric oxide (NO) pathway, and may therefore differentially affect the responses to phosphodiesterase 5 (PDE5) inhibitors and soluble guanylyl cyclase (sGC) stimulators. So far, the effects of the sGC stimulator riociguat on HPV have been poorly characterized.

**Materials and methods:**

Contraction was recorded in pulmonary arteries (PA) in a wire myograph. Anesthetized rats were catheterized to record PA pressure. Ventilation and perfusion were analyzed by micro-CT-SPECT images in rats with pulmonary fibrosis induced by bleomycin.

**Results:**

The PDE5 inhibitor sildenafil and the sGC stimulator riociguat similarly inhibited HPV *in vitro* and *in vivo*. Riociguat was more effective as vasodilator in isolated rat and human PA than sildenafil. Riociguat was ≈3-fold more potent under hypoxic conditions and it markedly inhibited HPV *in vivo* at a dose that barely affected the thromboxane A_2_ (TXA_2_) mimetic U46619-induced pressor responses. Pulmonary fibrosis was associated with V’/Q’ uncoupling and riociguat did not affect the V’/Q’ ratio.

**Conclusion:**

PDE5 inhibitors and sGC stimulators show a different vasodilator profile. Riociguat was highly effective and potentiated by hypoxia in rat and human PA. *In vivo*, riociguat preferentially inhibited hypoxic than non-hypoxic vasoconstriction. However, it did not worsen V’/Q’ coupling in a rat model of pulmonary fibrosis.

## Introduction

Pulmonary hypertension (PH) is a life-threatening progressive disorder of various aetiologies, exhibiting a complex pathophysiology characterized by vasoconstriction, vascular remodelling and thrombosis that lead to reduced pulmonary arterial lumen and elevated pulmonary arterial pressure (PAP) [1–3]. The World Health Organization (WHO) classifies PH into five groups. Currently therapies approved for pulmonary arterial hypertension (PAH; i.e. group 1 of PH) are directed mainly to reduce the vasoconstrictor component [1]. Recently, the soluble guanylyl cyclase (sGC) stimulator riociguat has been approved for both PAH and chronic thromboembolic pulmonary hypertension (CTEPH, i.e. group 4 of PH) [4]. Unfortunately, there are no approved specific therapies for the most common forms of PH: PH due to left heart disease (group 2) and associated to lung diseases and hypoxia (group 3, e.g. associated to chronic obstructive pulmonary disease [COPD] or idiopathic pulmonary fibrosis [IPF]). PH is a frequent complication of COPD [5] and IPF [6], present in more than half of the patients with severe disease. Despite the only moderate increase in PAP in these patients, the presence of PH is associated to worse prognosis [6–8].

HPV is a highly conserved adaptive physiological mechanism that increases pulmonary vascular resistance in poorly aerated lung regions, thereby reducing blood flow to the hypoxic alveoli, redirecting it to the best ventilated ones [9]. The sensor, transduction, and effector mechanisms involved in HPV reside essentially in the pulmonary arterial smooth muscle cells [9, 10]. A limitation of currently available vasodilators systemically administered is the uncoupling effects on ventilation-perfusion (V’/Q’) due to the inhibition of hypoxic pulmonary vasoconstriction (HPV) as described below [11].

Mild, moderate or severe hypoxemia is a common feature of PH associated to COPD, IPF or sleep apnea. In this scenario, HPV represents a crucial protective mechanism that redistributes blood flow away from diseased (hypoxic) lung tissue to the best oxygenated alveoli, reducing shunt flow to optimize oxygen saturation [9] at the expense of an elevated PAP. Systemic administration of vasodilators in PH associated with lung diseases might reduce PAP and improve the outcomes of these patients in terms of reducing right ventricular load and increasing exercise tolerance [8]. However, these drugs, by inhibiting HPV, may also increase blood flow to poorly-ventilated or non-ventilated areas of the lung, worsening preexisting V’/Q’ mismatch and hypoxemia. Therefore, the use of vasodilator therapy in COPD and IPF patients with PH is not currently recommended [1]. However, due to the poor prognosis of patients presenting PH associated to COPD/IPF and the lack of alternative treatments, some researchers have suggested the cautious use of vasodilators [12–17]. The phosphodiesterase 5 (PDE5) inhibitor sildenafil has demonstrated a significant reduction in pulmonary vascular resistance in patients with lung fibrosis [15] or COPD [14]. Larger studies in IPF patients have also shown a significant positive effect on V’/Q’ mismatch and arterial oxygenation while the effects on exercise capacity are inconsistent [16, 17]. In contrast, sildenafil worsened V’/Q’ coupling and impaired arterial oxygenation in patients with COPD [14]. In an open-label, uncontrolled pilot trial in patients with interstitial lung disease and PH, riociguat improved pulmonary vascular resistance, cardiac output and exercise capacity [12]. However, a subsequent randomized controlled trial in patients with PH associated with idiopathic interstitial pneumonias was prematurely terminated due to an apparent increase in death and serious adverse events (NCT02138825, Clinicaltrials.org). In addition, a small pilot study showed that acute riociguat reduced pulmonary pressure and vascular resistance and had no significant effect on arterial oxygenation [13]. Therefore, the treatment of group 3 PH with vasodilators would require further basic and clinical research.

Hypoxia is also a well-known modulator of the nitric oxide (NO)-cyclic GMP (cGMP) pathway. Because sGC stimulators and PDE5 inhibitors activate the NO-cGMP pathway in a different manner, we hypothesized that the two drugs may behave differently depending on the oxygen concentration. Thus, while PDE5 inhibitors prolong the action of cGMP, potentiating the effect of endogenous NO, stimulators of sGC such as riociguat may increase cGMP synthesis independently of NO. The effects of stimulators of sGC such as riociguat on HPV have hardly been investigated. In the present study, we analysed the effects of the sGC stimulator riociguat and the PDE5 inhibitor sildenafil on HPV in human and rat arteries *in vitro* an in rats *in vivo*.

## Methods

Forty one pathogen-free male Wistar rats (12 weeks of age) were obtained from Harlan Iberica (Barcelona, Spain). All rats were kept with free access to food and water throughout the whole experiment period and on standard rat chow and maintained at 24°C under a 12h light/12 h dark cycle. No animal died as a result of the experiments. Animals were sacrificed by an overdose of the anesthetic. All experimental procedures utilizing animals were carried out according to the Spanish Royal Decree 1201/2005 and 53/2013 on the Care and Use of Laboratory Animals and approved by the institutional Ethical Committees of the Universidad Complutense de Madrid (Madrid, Spain) and the University of Valencia (Valencia, Spain). After written informed consent, human neoplasia-free samples of lung tissue discarded by the pathologist were obtained from 14 adult patients of both sexes (12 men and 2 women, 66 ± 3 years) undergoing lung carcinoma surgery. The procedures were approved by the Human Research Ethics Committee of the Hospital Universitario de Getafe and the regional Committee for Laboratory Animals Welfare.

### Vascular reactivity *in vitro*

Rat or human pulmonary artery (PA) and rat mesenteric artery (MA) rings (0.4–0.8 mm of internal diameter) were mounted in Krebs solution in a myograph [18, 19]. Arteries were exposed to KCl (80 mM), then 5-HT (10 μM) and Ach (1 μM) was added to test endothelial function. In some experiments arteries were mounted in 21% O_2_ and then exposed to hypoxia 0% O_2_ (2–3% final concentration of O_2_ in the Krebs solution) to elicit HPV and after returning to 21% O_2_, the TXA_2_ mimetic U46619 was added to the bath. Some arteries, incubated either in 0, 21 or 95% O_2_, were exposed a cocktail of three vasoconstrictors at submaximally effective concentrations (≈50% of their individual maximal response), i.e. U46619 at 30 nM, ET-1 at 3 nM and 5-HT at 3 μM and then a concentration response curve was constructed by cumulative addition of the sGC stimulators riociguat and BAY412272 and the PDE5 inhibitors sildenafil and tadalafil (all from 10^-9^M to 10^-5^M).

### Hemodynamic measurements

Rats were anesthetized (80 mg/kg ketamine and 8 mg/kg xylacine i.p.), tracheostomyzed and ventilated with room air (tidal volume 9 mL/kg, 60 breaths/min, and a positive end-expiratory pressure of 2 cm H_2_O, Nemi Scientific Inc, Medway, USA). Arterial saturation (SpO_2_) was continuously recorded using a pulsioxymeter (StarrOx) placed in the lower extremity of the animal. The carotid artery was cannulated for systemic arterial pressure (SAP) recording. After sternotomy, a catheter was placed in the PA through the right ventricle for PA pressure recording. Drugs were administered intravenously. Hypoxia was induced by ventilating the animals with a FIO_2_ of 0.1 for 5 min.

**Fig 1:**
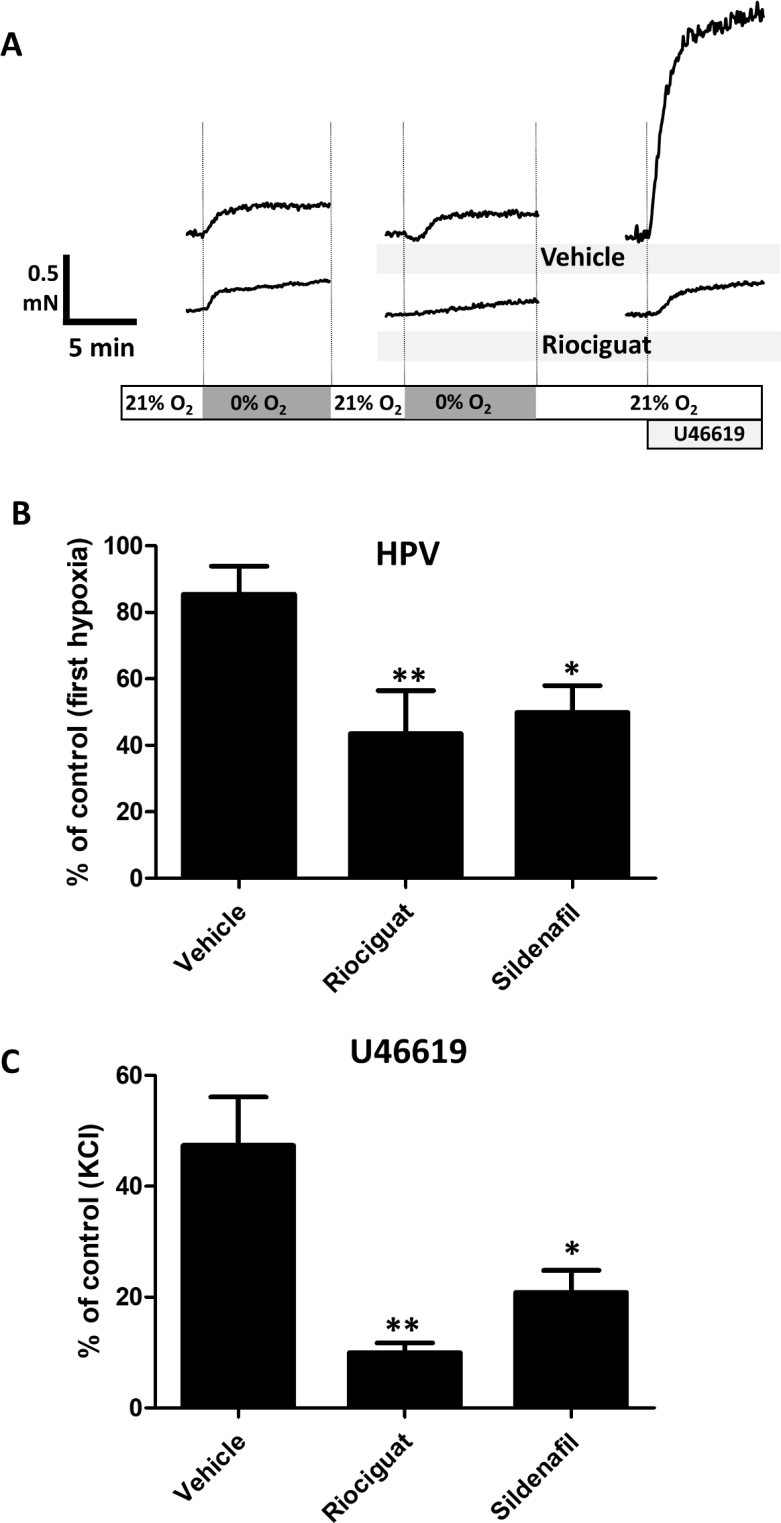
Riociguat and sildenafil inhibit hypoxic pulmonary vasoconstriction and U46619-induced vasoconstriction in isolated rat pulmonary arteries (PA). PA bubbled with 21% O_2_, 74% N_2_ and 5% CO_2_ were initially exposed to hypoxia (95% N_2_ and 5% CO_2_), then treated with the drugs (both at 100 nM), exposed again to hypoxia and finally treated with U46619 (100 nM). Panel A shows the study protocol and representative traces. Panels B and C show the results (means ± SEM, n = 12, 9 and 8 for vehicle, riociguat and sildenafil, respectively) for the effects of hypoxia and U46619, respectively. *P<0.05 and **P < 0.01 versus vehicle.

The experimental design for the acute in vivo study is shown in Fig 1. After a 10 minutes stabilization period under normoxic (room air) ventilation, rats were exposed to global hypoxia (FIO_2_ of 0.1, I, GH) for 5 minutes followed by a 20 minutes recovery period of normoxia. Then vehicle (dimethyl sulfoxide, DMSO, 500 μl, n = 6), riociguat (0.1 mg/kg, n = 6) or sildenafil (0.5 mg/kg, n = 6) were infused manually over a period of 5 minutes through the jugular vein, and after 40 minutes rats were again exposed to GH (II). Finally rats were treated with U46619 (U4) (0.003 mg/kg) for 5 minutes. At the end the animals were euthanized by stopping the ventilator.

### Bleomycin model of pulmonary fibrosis

Rats were anaesthetized with ketamine/medetomidine and then a single dose of bleomycin at 3.75 U/kg (dissolved in 200 μL of saline) was administered intratracheally via the endotracheal route [20]. This dose of bleomycin reproducibly generated pulmonary fibrosis in previous experiments [21]. Sham control treated rats received the identical volume of intratracheal saline instead of bleomycin. The rats were weighed and observed daily for any signs of severe suffering or distress (e.g. inability to rise or ambulate, weight loss >20%, labored breathing, distended abdomen, hunched posture and ruffled fur), which did not happen in any animal and there was no need for euthanasia.

### Analysis of ventilation and perfusion by micro-CT-SPECT images

*In vivo* imaging and quantitative analysis were performed using computer tomography (micro-CT) and single photon emission computed tomography (Albira micro-CT-SPECT-PET Imaging System (Oncovision®, Spain) using pinhole collimators and a radius of rotation of 3.5 cm [22]. Perfusion lung imaging depends on the embolization of 99^m^Tc-labelled 10–40 μm human albumin macroaggregates (MAA-Tc99^m^) and ventilation is monitored using diethylene-triamine-pentaacetate (DTPA-Tc99^m^) (Molipharma, Valencia, Spain) (10 mCi) [23]. Twenty-one days after the administration of bleomycin or saline, a single 0.1 mg·kg−1 dose of riociguat or vehicle was administered intraperitoneally. Rats were tracheally intubated through the oral cavity after anesthetization with ketamine and xylazine (90 and 6 mg/kg, respectively). The animals were then ventilated (0.02 L/min, 125 strokes/min) on a rodent ventilator (model 683; Harvard Apparatus) with DTPA-Tc99^m^ for 15 min. After DTPA-Tc99^m^ delivery, the animals were removed from the ventilator and allowed to breathe freely. After the ventilation SPECT scan, rats were injected with 0.5–1 mCi of MAA-Tc99^m^ via the tail vein. The relationship between ventilation and perfusion data was determined with PMOD™ software analyzing the intensity of radiation (arbitrary units) of each volume of interest (VOI) of the whole lung region selected of 256 image sections for each animal study corrected by the maximal activity. Corrected radiation intensities of ventilation (V) and perfusion (Q) studies were represented.

### Drugs

All drugs were from Sigma-Aldrich (Spain) except tadalafil (SantaCruz Biotechnology, USA) and riociguat (MedChem Express, China)

### Statistical analysis

Results are expressed as means ± SEM. N indicates the number of animals or arteries tested. Individual cumulative concentration-response curves were fitted to a logistic equation. The drug concentration exhibiting 50% of the maximal effect was interpolated from the fitted concentration-response curves for each ring and expressed as negative log molar concentration (pIC_50_). Statistical analysis was performed using a one-way ANOVA followed by Bonferroni *post hoc* test or paired Student’s t test as appropriate. Differences were considered statistically significant when P<0.05.

## Results

### Effects on hypoxia- and U46619-induced PA contractions *in vitro*

In isolated PA, bubbling the myograph chamber with nominally O_2_-free (95% N_2_-5% CO_2_) yields a 2–3% concentration of O_2_ in the Krebs solution. In the absence of any other vasoconstrictor stimuli, shifting from normoxia (21% O_2_) to hypoxia induced a contractile response (HPV). As shown in Fig 1A, this contraction was small (i.e. ≈5–10% of the maximal response induced by 80 mM KCl), sustained for 10 min, reversible after returning to normoxia and reasonably well reproduced after a period of 20 min of normoxia in the presence of vehicle (86±8% of the first response). Stronger responses to hypoxia can be achieved if arteries are primed with a pre-tone induced by a vasoconstrictor, but then the effects of any subsequent intervention can be attributed to either the hypoxic response or the pre-tone. After returning again to normoxic conditions for 10 min, exposure to the thromboxane A_2_ mimetic U46619 (100 nM) leads to a sustained contractile response. Riociguat and sildenafil inhibited HPV (Fig 1). At a concentration (100 nM for both drugs) selected to produce a similar inhibitory effects on HPV (50±15% and 58±9% of the response in vehicle-treated arteries, respectively, Fig 1B), the response to U46619 was more strongly inhibited by riociguat (21±4% and 43±8%, respectively, p<0.05 sildenafil vs riociguat, Fig 1C).

### Vasodilator responses in pulmonary and mesenteric arteries. Effects of oxygen

In order to compare the vasodilator responses *in vitro* under different conditions of oxygenation and for a wide range of concentrations of the drugs, we continuously exposed rat and human PA to nominally 0, 21 or 95% oxygen. The endothelial function was tested with 1 μM acetylcholine (ACh) in arteries pre-contracted by 5-HT (10 μM). Because vasodilator responses are known to vary widely depending on the vasoconstrictor stimuli, arteries were contracted with a mixture of three vasoconstrictors, or its analogs, which are known to be elevated in PH (the thromboxane A_2_ analog U46619, ET-1 and 5-HT). This pan-constrictor cocktail induces a strong and sustained vasoconstrictor response (similar to that induced by 80 mM KCl). The potency of the vasodilator drugs under the different conditions was calculated as the concentration producing 50% relaxation (expressed as the negative log molar, pIC_50_) and the efficacy as the maximal relaxation (E_max_, %) induced by the highest concentration of the drug tested (Table 1).

**Table 1:** Potency and efficacy of sGC stimulators in rat and human arteries (calculated from data shown in Figs 2 and 3).

	PA 21% oxygen			PA hypoxia			PA 95% oxygen			MA 95% oxygen		
	pIC_50_	E_max_ (%)	n	pIC_50_	E_max_ (%)	n	pIC_50_	E_max_ (%)	n	pIC_50_	E_max_ (%)	n
Riociguat (rat)	6.88 ± 0.24	82 ± 2	7	7.39 ± 0.09 *	95 ± 4	6	6.86 ± 0.04	89 ± 2	6	7.45 ± 0.08 *	108 ± 6 **	6
Riociguat (human)	n.d.	n.d.	-	6.60 ± 0.19	77 ± 5	11	6.07 ± 0.25	65 ± 9	10	n.d.	n.d.	-
BAY412272 (rat)	n.d.	n.d.	-	7.87 ± 0.16	111 ± 4	5	7.52 ± 0.21	94 ± 9	6	7.59 ± 0.19	98 ± 4	5

PA, pulmonary arteries; MA, mesenteric arteries. pIC_50_, drug concentration inducing 50% relaxation (expressed as negative log molar), E_max_ maximal relaxation achieved. Results are means ± SEM

* p < 0.05 and

** p < 0.01 vs PA 95% oxygen

n.d., not determined.

**Fig 2:**
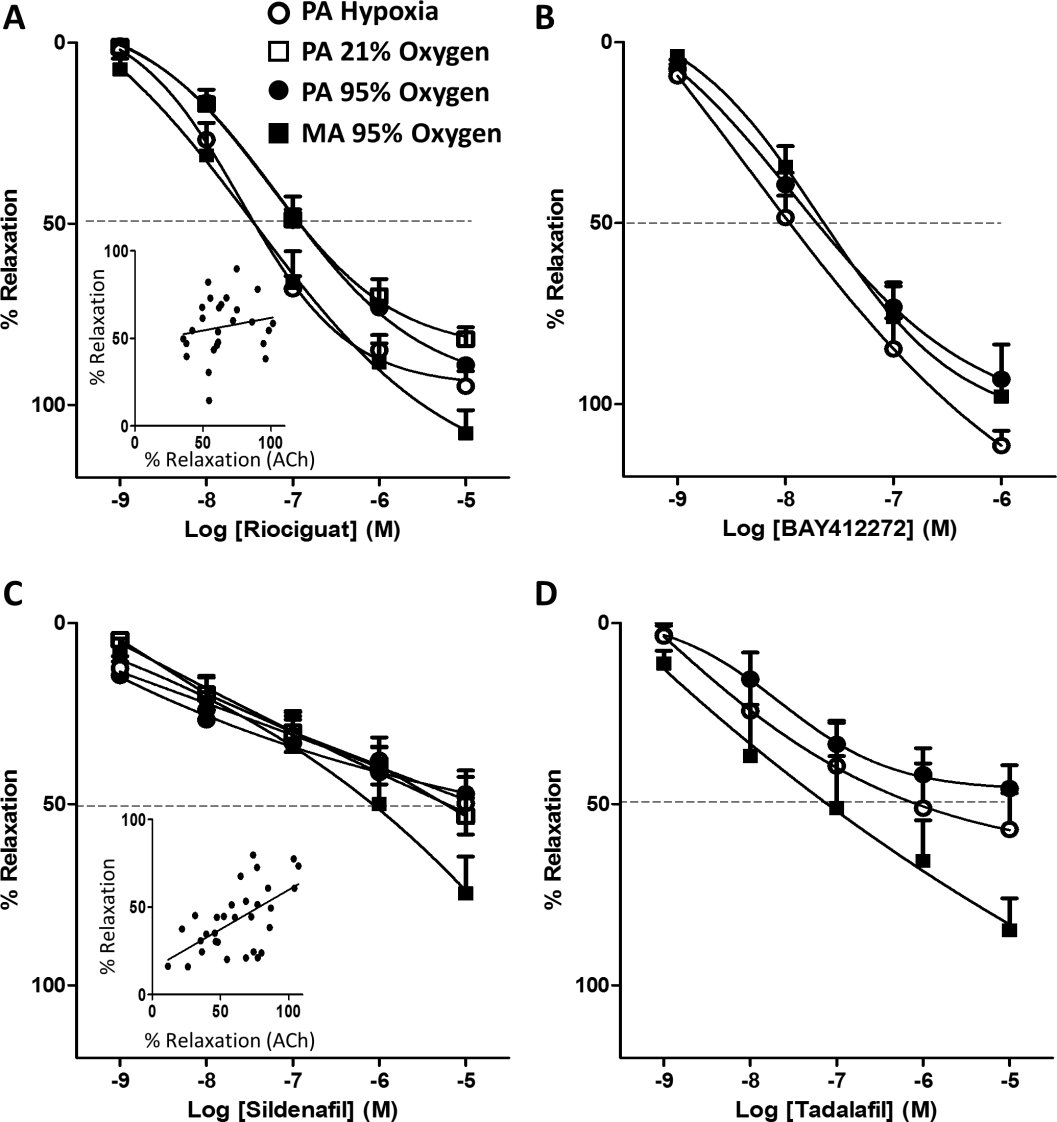
Oxygen and pulmonary selectivity of the vasodilator effects in isolated rat arteries. The sGC stimulators riociguat (A) and BAY412272 (B) and the PDE5 inhibitors sildenafil (C) and tadalafil (D) relax rat pulmonary (PA) and mesenteric arteries (MA) bubbled with 21% O_2_-74% N_2_-5% CO_2_, 95% O_2_-5% CO_2_ or with 95% N_2_-5% CO_2_ (hypoxia). Arteries were initially stimulated with a cocktail of U46619 (30 nM), 5-HT (3 μM) and endothelin (3 nM) and, thereafter, the drugs were added in a cumulative fashion (all from 10^-9^M to 10^-5^M). The insets show the correlation between the initial response to ACh (1 μM) and the subsequent relaxation induced by riociguat (0.1 μM) or sildenafil (1 μM) in each artery (data from MA and PA under all conditions). Results are means ± SEM (n = 7, 6, 6 and 6 for riociguat 7, 9, 9 and 5 for sildenafil, in PA at 21, 95, and 0% O_2_ or MA, respectively, and 4, 4 and 4 for tadalafil and 6, 5 and 5 for BAY412272 in PA at 95, and 0% O_2_ or MA, respectively). The parameters calculated from these curves and their statistical analysis are shown in Table 1.

Cumulative addition of riociguat to rat PA (Fig 2A) produced a concentration-dependent relaxation which was 3.1 fold less potent (p < 0.05) under 21 or 95% oxygen than under hypoxic conditions. Riociguat was highly effective, at 10 μM it induced a near maximal relaxation under high oxygen or hypoxic conditions. Riociguat also induced a highly effective relaxation in rat MA, being 3.6 more potent (p<0.05) than in PA (both at 95% O_2_). A qualitatively similar pattern in PA was observed for BAY412272, another sGC stimulator (Fig 2B), but the 2.2 fold difference in potency under high vs low oxygen was not statistically significant. The relaxant effect of BAY412272 in MA was very similar to that in PA under high oxygen. The PDE5 inhibitors sildenafil and tadalafil also produced concentration-dependent relaxations (Fig 2C and 2D) which were not significantly different under 0, 21 or 95% oxygen in rat PA. When compared under the same conditions of high oxygen, the maximal relaxations of PDE inhibitors were stronger in MA than in PA. Notably, the two PDE5 inhibitors were much less effective than the sGC stimulators in PA, with the maximal response achieved at the highest concentration tested (10 μM) being around 50% in all conditions and, therefore, pIC_50_ could not be properly calculated. In addition, the curves for the PDE5 inhibitors had a milder slope. The insets in Fig 2 plot the correlation between the vasodilator response to ACh (i.e. the endothelial function) and the relaxation induced in each ring by the concentration of the drugs producing ≈50% relaxation under all conditions studied. While no correlation was observed for riociguat, the relaxant response to ACh in each arterial ring predicted the vasodilator effect of sildenafil (slope = 0.49 ± 0.12, R^2^ = 0.38, p < 0.05).

**Fig 3:**
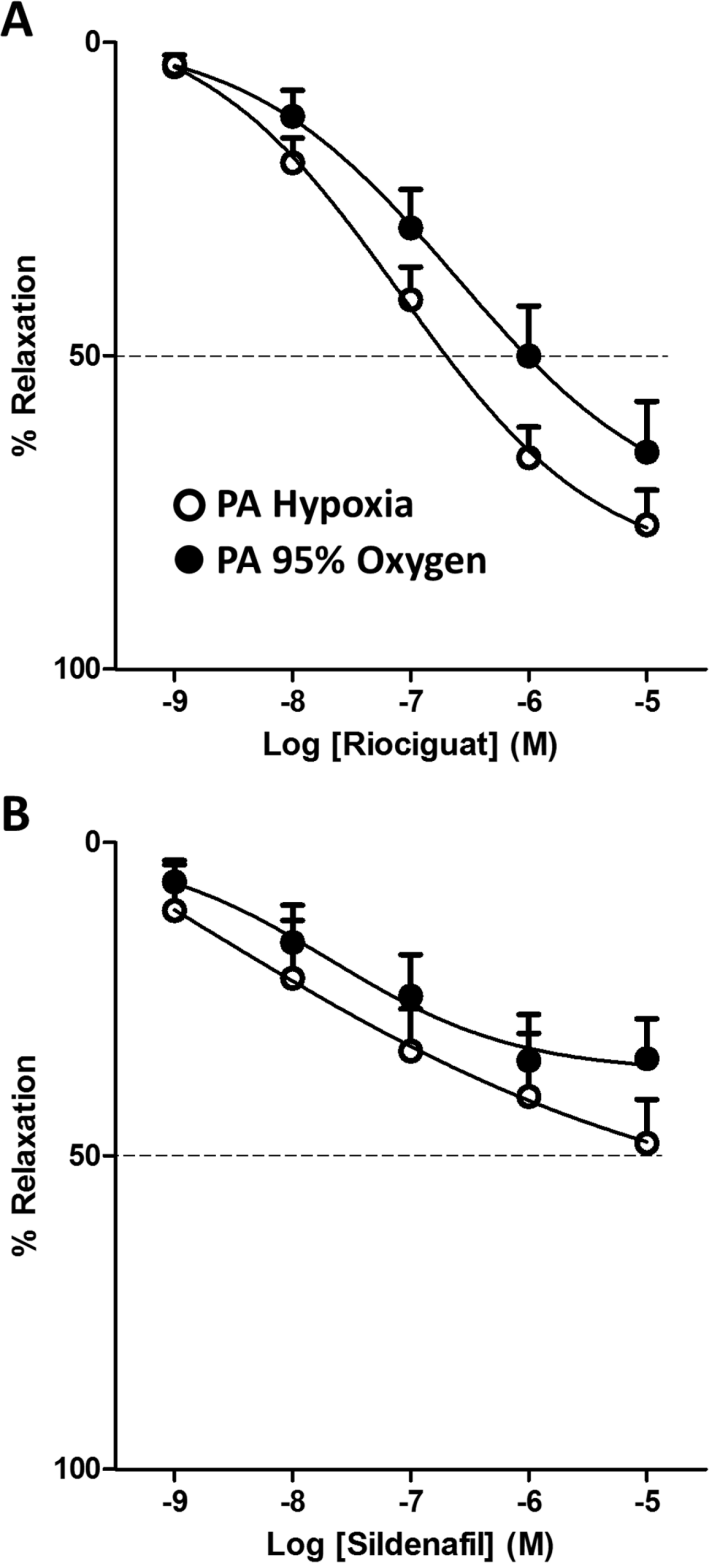
Oxygen selectivity of the vasodilator effects in isolated human arteries. Riociguat (A) and sildenafil (B) relax human pulmonary arteries (PA) bubbled with 21% O_2_-5% CO_2_-74% N_2_, 95% O_2_-5% CO_2_ or with 95% N_2_-5% CO_2_ (hypoxia). Arteries were initially stimulated with a cocktail of U46619 (30 nM), 5-HT (3 μM) and endothelin (3 nM) and, thereafter, the drugs were added in a cumulative fashion (all from 10^-9^M to 10^-5^M). Results are means ± SEM (n = 11 and 10 for riociguat and 7 and 6 for sildenafil). The parameters calculated from these curves and their statistical analysis are shown in Table 1.

In human PA riociguat also tended to be more potent under hypoxic than under high oxygen conditions (3.4 fold, p = 0.06, Fig 3). Riociguat was again more effective than sildenafil in both high and low oxygen, i.e. the maximal relaxation induced by the latter was less than 50%. When the vasodilator effects were compared in rat vs human PA (Fig 3 vs Fig 2), riociguat was 5–10 fold less potent (i.e. lower pIC_50_, p< 0.05) and less effective (i.e. lower E_max,_ p<0.05) in human PA.

### Pulmonary and systemic effects *in vivo*

**Fig 4:**
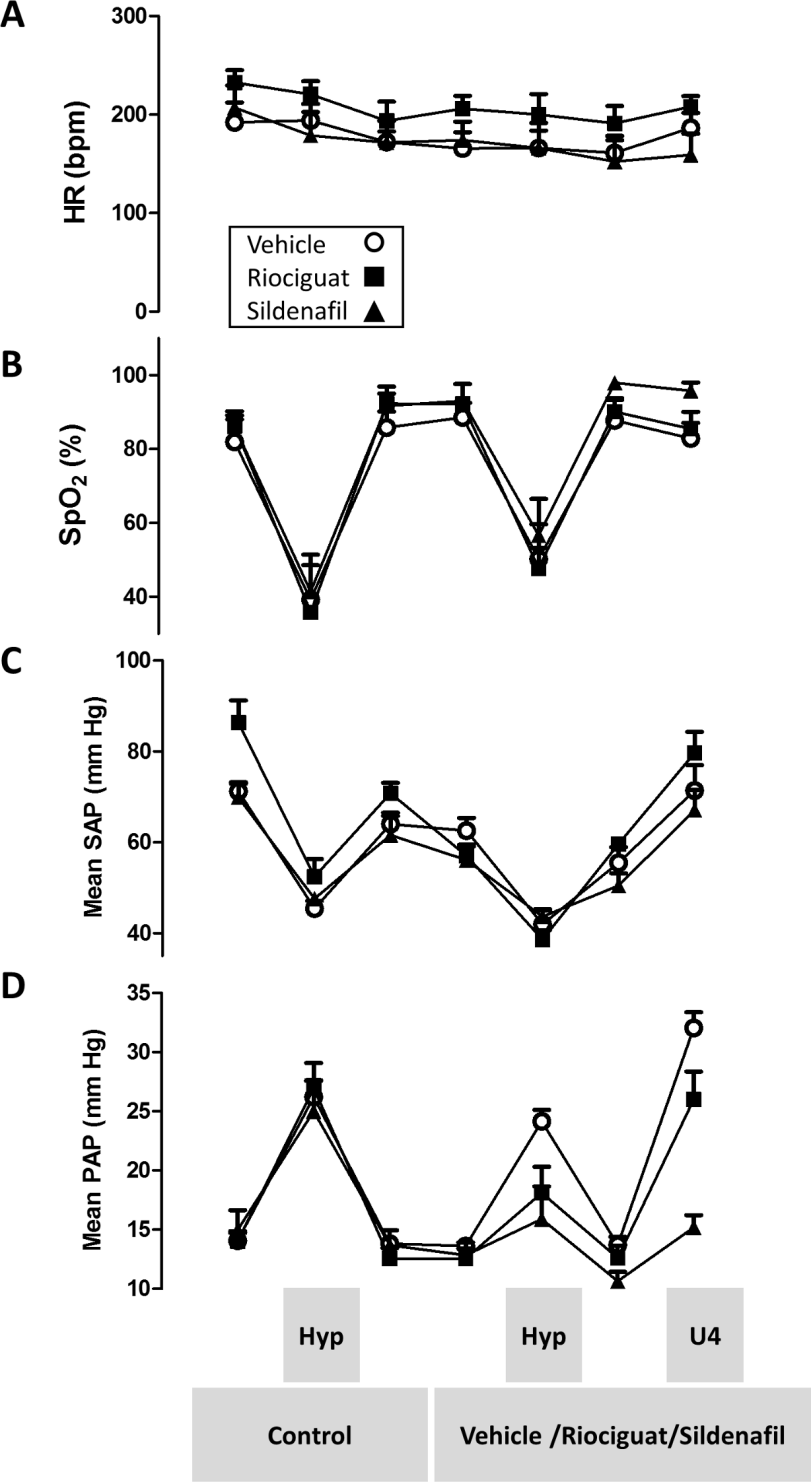
Effects of riociguat and sildenafil on systemic and pulmonary arterial pressure (SAP and PAP) and oxygen arterial saturation (SpO_2_). After stabilization, rats were ventilated with FIO_2_ = 0.1 (Hyp), then vehicle, riociguat (0.1 mg/kg) or sildenafil (0.5 mg/kg) were injected IV and after 20 min again ventilated with FIO_2_ = 0.1 and finally U46619 (0.003 mg/kg, U4) was injected IV. (A) Heart rate (HR), (B) SpO_2_, (C) SAP and (D) PAP. Results are means ± SEM (n = 6 for vehicle and 5 for riociguat and sildenafil). The graphs show absolute values. Relative changes and the statistical analysis of these results are shown in Fig 5.

**Fig 5:**
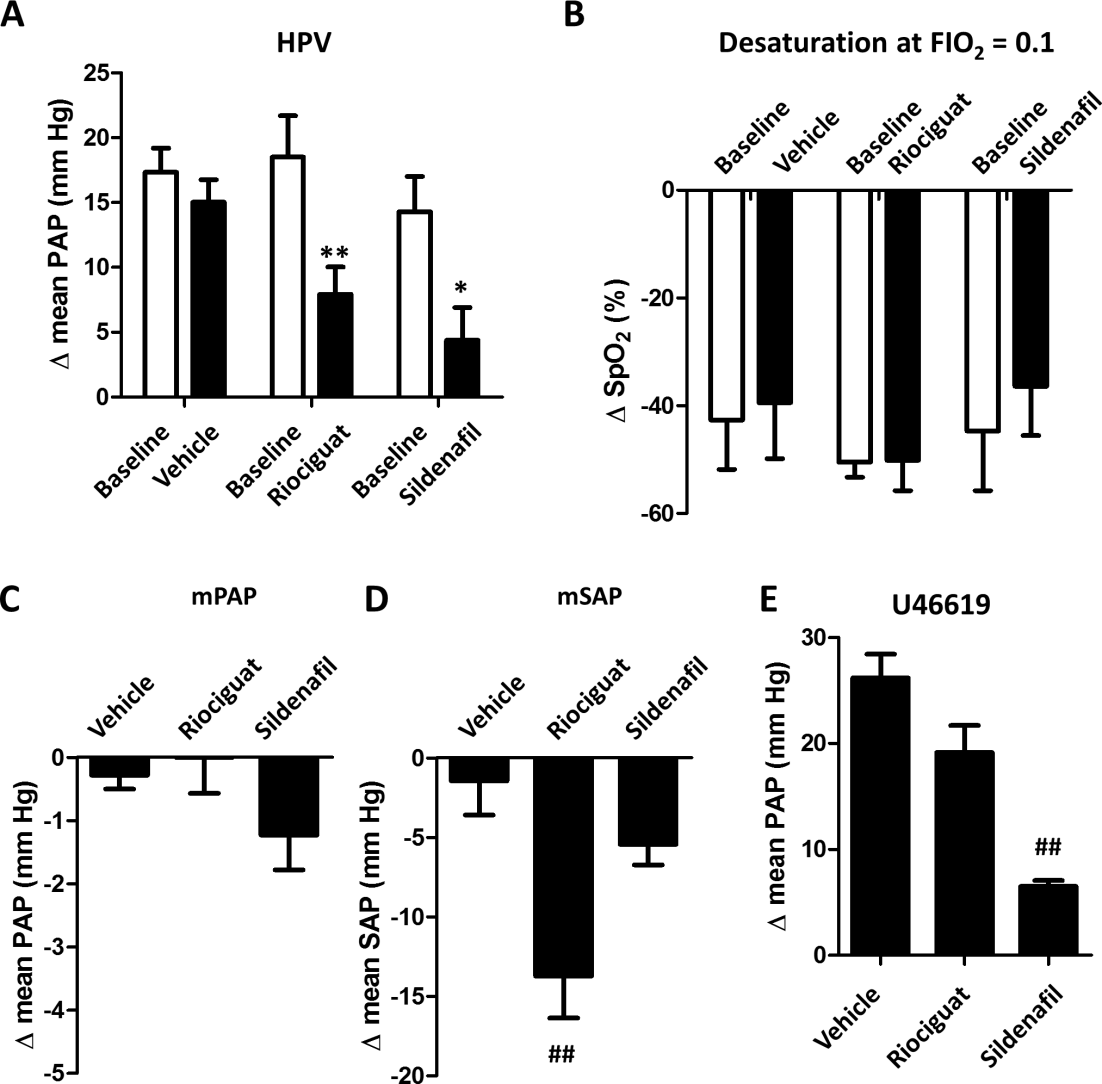
Changes in systemic and pulmonary arterial pressure and oxygen arterial saturation. Changes in mean PAP (panel A, HPV) and in saturation (panel B, SpO_2_) in response to hypoxia before (baseline) or after the exposure to the drugs. Changes in mean pulmonary (C) and systemic (D) pressure induced by the drugs. Effects on the vasopressor response to U46619 (E). Data are calculated from experiments shown in Fig 4, *p<0.05 and **p < 0.01 versus baseline (paired t test), ## p< 0.01 vs vehicle (one-way Anova followed by Bonferroni’s test).

In order to analyze the effects *in vivo*, we employed a protocol with a repeated 5 min challenge to hypoxia (FIO_2_ = 0.1) before and after the exposure to the drugs. Baseline parameters were not significantly different among groups despite a trend for higher SAP in the experimental series of riociguat. Hypoxia led to an increase in PAP (≈8–12 mmHg, i.e. HPV), a decrease in SAP and a decrease in oxygen saturation with minimal and inconsistent effects on heart rate (Fig 4 shows the absolute values and Fig 5 the changes induced by each intervention). The administration of vehicle (DMSO) had no significant effect on PAP or SAP and did not modify the changes induced by hypoxia (Figs 4 and 5). Riociguat and sildenafil were administered at doses selected in preliminary experiments to produce similar inhibitory effects on HPV (0.1 and 0.5 mg/kg, respectively). This doses produced minimal changes in PAP but riociguat significantly reduced SAP. However, it should be noted that there is a mild but progressive decrease in SAP along the experimental period in all groups which is more prominent in the riociguat group, probably due to higher baseline levels. Upon exposure to hypoxia, both drugs inhibited the increase in PAP to a similar extent but they did not modify the desaturation. Riociguat and sildenafil had no significant effect on heart rate, pulmonary arterial dP/dt_max_ or the systemic hypotension induced by hypoxia. Finally, sildenafil strongly inhibited the pulmonary pressor response to U46619, while the partial inhibition by riociguat was mild and borderline significant (p = 0.06).

### Effects on ventilation-perfusion in rats with pulmonary fibrosis

**Fig 6:**
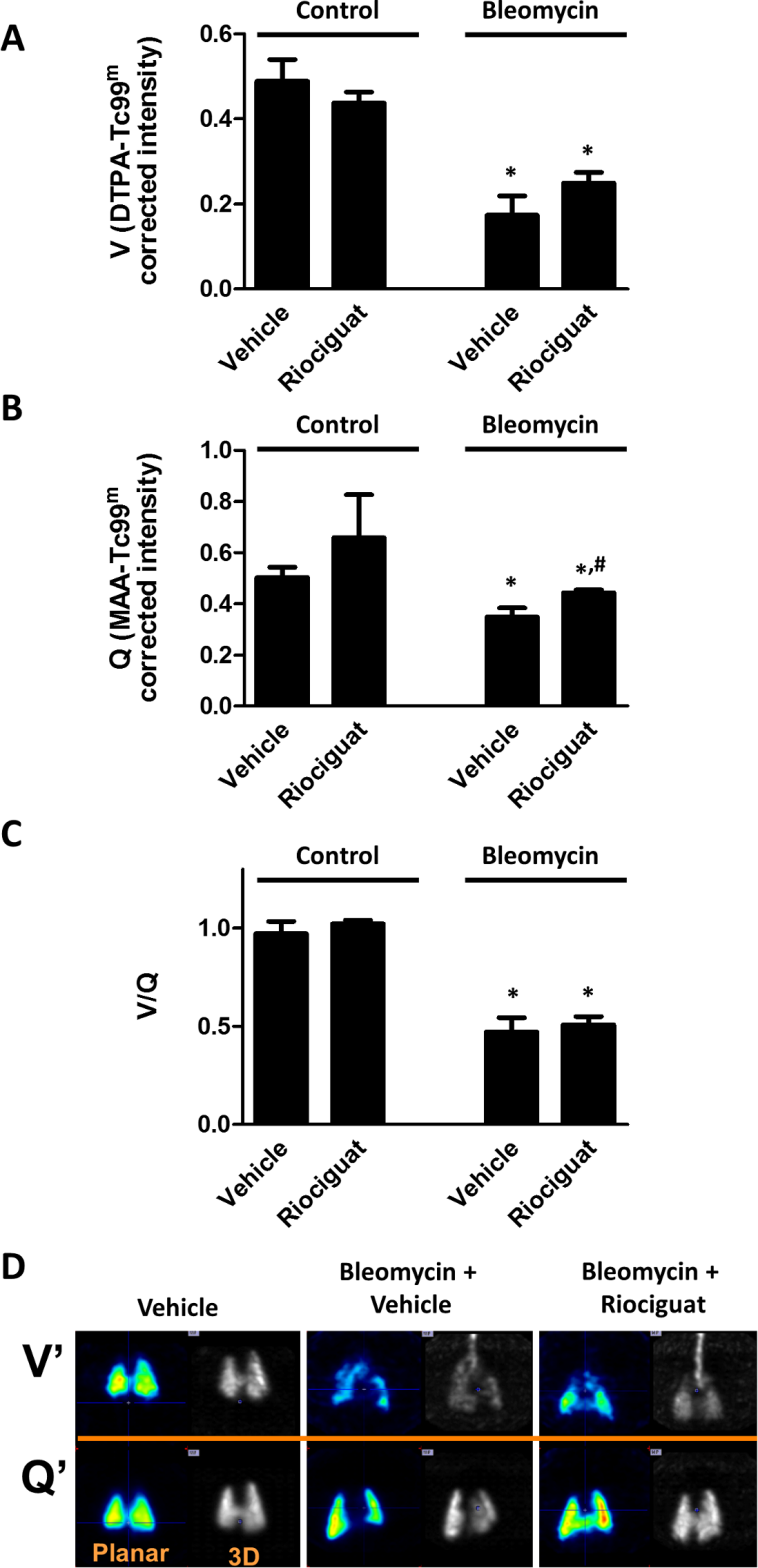
Effects of riociguat on ventilation-perfusion coupling (V’/Q’). Effects of riociguat on (A) ventilation (V’), (B) perfusion (Q’) and (C) ventilation-perfusion coupling (V’/Q’) in control and bleomycin-induced fibrotic rats. (D) Representative images of ventilation and perfusion were obtained by micro-CT-SPECT. A single dose of riociguat (0.1 mg/kg) or vehicle was administered 21 days after the administration or bleomycin or saline. The relationship between the ventilation and perfusion data was determined using PMOD™ software to analyze the intensity of radiation (arbitrary units) of each volume of interest. Results are means ± SEM (n = 8). *P<0.05 vs vehicle control and #P < 0.05 versus vehicle bleomycin.

In order to analyze the effects of the drugs on ventilation and perfusion we used control rats and a model of bleomycin-induced fibrosis in which V’/Q’ is strongly unpaired. Three weeks after bleomycin or vehicle administration, ventilation and perfusion were analyzed by micro-CT-SPECT imaging. In control animals, acute administration of riociguat had no significant effects on ventilation, perfusion or the V’/Q’ ratio (Fig 6). Fibrosis induced a strong decrease in both ventilation and perfusion resulting also in reduced V’/Q’. Acute administration of riociguat had no significant effect on ventilation, produced a modest but significant increase in perfusion and again did not modify V’/Q’. These data resemble those recently reported for sildenafil in the same model and identical conditions, in which sildenafil had no significant effect on ventilation, perfusion or V’/Q’ [22].

## Discussion

Herein we show that PDE5 inhibitors and sGC stimulators show a different vasodilator profile. Riociguat was highly effective, not correlated with endothelial function and potentiated by hypoxia in rat and human PA. *In vivo*, riociguat preferentially inhibited hypoxic than non-hypoxic vasoconstriction. However, it did not worsen V’/Q’ coupling in rat model of pulmonary fibrosis.

NO exerts most of its actions, including vasodilation, by binding to and activating sGC, resulting in increased intracellular cGMP levels. In order to increase the defective NO signaling pathway in PH, two different therapeutic approaches have been developed: to increase cGMP synthesis by increasing sGC activity and to prevent cGMP degradation by inhibition of PDE5. In addition to NO and NO releasing compounds, two classes of drugs have been developed to increase cGMP synthesis, sGC stimulators and sGC activators [24]. sGC stimulators, such as riociguat, sensitize sGC to low levels of bioavailable NO but can also increase sGC activity in the absence of NO. In contrast, sGC activators preferentially activate sGC when it is in an oxidized NO-insensitive form, but its therapeutic use has not been approved yet. Thus, stimulation of sGC may have a theoretical advantage over PDE5 inhibition because of its NO-independent mode of action [4]. Therefore, sGC stimulators and activators could still be effective when NO levels are impaired, as occurs in PH. On the contrary, PDE5 inhibitors may be more effective when PDE5 is overexpressed as it has been shown in patients with IPF and PH [22]. The present experiments in isolated arteries clearly demonstrate this concept. In PA the maximal relaxant response of sildenafil was only 50% (Fig 2C) while riociguat was able to induce a full relaxant response (Fig 2A). This difference was reproduced in human PA (Fig 3) an in rat PA by the sGC stimulator BAY412272 and the PDE5 inhibitor tadalafil (Fig 2B and 2D), demonstrating that it is a characteristic feature of these drug classes and conserved between species. This difference presumably reflects a low basal NO activity under these conditions so that even after a strong inhibition of PDE5 there is insufficient cGMP to induce a full relaxation. In fact, we found that the NO-dependent endothelial function (relaxation to ACh) predicts well the response to PDE5 inhibitors but does not affect the response to sGC stimulators.

The systemic effects of oral and intravenous vasodilators leading to severe hypotension may preclude the use of effective doses of these drugs. The development of pulmonary selective drugs is a long-lasting unmet need [25, 26]. The results of the present study show that both PDE5 inhibitors and sGC stimulators display no pulmonary selectivity *in vitro*, showing similar or stronger effects in MA than in PA. *In vivo*, sildenafil and riociguat had weak effects on basal PAP, but reduced basal systemic arterial pressure, which was particularly evident for riociguat as previously reported [27]. In contrast, the pressor effects of U46619 were inhibited by sildenafil in the pulmonary circulation but unaffected in the systemic one, showing a clear pulmonary selectivity under these conditions which is consistent with previous studies with the drug in acute pulmonary hypertension induced by U46619 [26]. These differences in the systemic and pulmonary circulation reflect a different role of the NO-cGMP-PDE5 signaling pathway in the systemic and pulmonary circulation and a different regulation by thromboxane receptors in both vascular beds.

Hypoxia is also a well-known modulator of the NO pathway [28] and may therefore differentially affect the responses to PDE5 inhibitors and sGC stimulators. We have compared the effects of sildenafil with the novel sGC stimulator riociguat under well controlled conditions *in vitro* in rat and human arteries. Several studies have described the inhibitory effects of PDE5 inhibitors on HPV [29–32] while the effects of riociguat have been hardly analyzed [33]. Herein we show that the sGC stimulator riociguat shows a similar profile to sildenafil in inhibiting HPV *in vitro*, *i*.*e*. in isolated pulmonary arteries, and *in vivo*, *i*.*e*. the pulmonary pressor response to hypoxia. We compared their inhibitory effects on HPV to those on the vasoconstrictor response to the thromboxane A_2_ analog U46619. An ideal drug should inhibit U46619-induced responses at doses that preserve HPV. *In vitro*, both drugs were at least as effective on U46619-induced contractions as on HPV. However, *in vivo*, riociguat, at a dose which markedly inhibited HPV or induced systemic hypotension, hardly affected the U46619-induced pressor responses while sildenafil strongly inhibited it.

Thus, we analyzed the influence of oxygen on the vasodilator responses over a wide range of concentrations of the drugs. We chose a cocktail of vasoconstrictors, a thromboxane analog, ET-1 and 5-HT, inducing a strong contractile response under high, normal or low oxygen concentrations. The rationale for this protocol is that these are the main vasoactive factors known to be involved in PH [3] and that the possible differences in the potency or efficacy of the vasodilators depending on the vasoconstrictor stimuli can be minimized. We found that riociguat induced a 3-fold stronger vasodilator effects under hypoxic vs normal or high oxygen conditions in both human and rat PA, while these differences were smaller for the other sGC stimulator studied BAY412272 (2-fold). In contrast, the effects of sildenafil or tadalafil were similar in high, normal or low oxygen.

A limitation of our *in vitro* study is that we evaluated the effects of the drugs in healthy rat or human arteries or *in vivo* in healthy rats. To analyze a possible impact in a more clinically relevant context of V’/Q’ mismatch, we studied the effects of the drugs in a rat model of IPF associated with PH induced by bleomycin. This model is characterized by elevated right ventricular systolic pressure (≈ 45 mm Hg), right ventricular hypertrophy and vascular remodelling [34]. Ventilation and perfusion analysis, as monitored by the analysis of the inhaled DTPA-^99m^ Tc signal and perfused MAA-^99m^ Tc [22, 35]. Rats showed a markedly impaired V’/Q’ SPECT ratio three weeks after bleomycin-induced pulmonary fibrosis. Acute administration of sildenafil [22] and riociguat (present data) had no effect on ventilation in the bleomycin-induced pulmonary fibrosis model associated with PH, riociguat (but not sildenafil) induced a modest increase in lung perfusion but there were no significant changes in the V′/Q′ ratio. Several other limitations should be mentioned in our study. First, the hemodynamic study was conducted in open chest ventilated rats under anesthesia. Second, in the *in vivo* models, we have only tested a single drug dose. Third, despite the frequent use of the bleomycin model of pulmonary fibrosis, it has significant limitations in its ability to mimic human IPF since many treatments successful in the bleomycin model were not transferable to human IPF.

Oxygen-selective drugs which exert vasodilator effects preferentially in high ventilated/oxygenated lung regions when administered systemically are currently lacking but they could represent a significant therapeutic advance, particularly in patients with PH associated to hypoxia and/or V/Q mismatch.

In conclusion, as a result of different mechanisms of action, PDE5 inhibitors and sGC stimulators show a different vasodilator profile. Both sildenafil and riociguat effectively inhibit HPV *in vitro*. *In vivo*, riociguat, at a dose which markedly inhibited HPV hardly affected the U46619-induced pressor responses while sildenafil similarly inhibited hypoxic and non-hypoxic vasoconstriction. This may be due to a 3-fold stronger vasodilator potency of riociguat under hypoxic conditions in both human and rat PA. However, the two drugs did not worsen V’/Q’ coupling in rat model of IPF.
